# Case of Pyromania

**Published:** 1850-07-01

**Authors:** 


### CASE OF PYROMANIA.

At the end of August and beginning of September, 1849, several incendiary conflagrations occurred in the Commune of Grillon (Vaucluse). It seems that the inhabitants of the village were in the habit of thrashing their corn in a field, belonging to one B., and that they afterwards stacked the straw on the same spot. In the short space of eighteen days, no less than thirteen stacks were burned in succession. The fire seemed on each occasion to commence in the centre of the stack, and commonly broke out about three P.M. The village authorities, unable to solve the mystery, requested some assistance, and M. de Vérot, procureur at Orange, accompanied by another magistrate, arrived to help them. Whilst these functionaries were engaged in making some preliminary inquiries, a young girl, aged fourteen, daughter of the man in whose field all the fires had happened, requested an interview, and on coming before them, exclaimed in Provençal, "*Mousu aie trouva la besougne,*" ("Gentlemen, I have found out the matter,") at the same time handing to them a piece of reed, six or seven inches long. On examination, the reed was found to be filled with coarse gunpowder; it was stopped at one end, and had two lucifer-matches firmly fixed in the other. The young girl declared that she had found the object concealed in a heap of straw on the field. There was something in the girl's manner and story which excited the suspicions of the magistrates, so they instituted a strict search over her father's house, and found some pieces of cut reed, some coarse gunpowder, similar to that in the reed, and a box of lucifers, from which only two matches were missing, the lid of which had black finger-marks upon it. They then proceeded to interrogate the girl herself, whom they found exceedingly quick and intelligent; but she adhered to her story, and firmly denied knowing anything about the construction of the tube. However, after some days she grew tired of the repeated examinations, and confessed that she alone had prepared the tube; that she alone had set fire to the thirteen stacks; that frequently, about three P.M., an impulse, which she could not control, urged her, with irresistible force, to go and set fire to the straw; that, nevertheless, she had taken every precaution to avoid being discovered; that after she had forcibly pushed the tube into the stack, she ran away trembling and agitated, and was frequently obliged to lie down on the ground in a state of extreme exhaustion. She added, that when the fire broke out, she went and hid herself, so as not to see it.

She was placed in confinement, and a commission of medical men appointed to examine into the state of her mind.

They report that they cannot discover anything abnormal in her condition; but, as yet, they have not given their final opinion, and the girl still remains under observation.

